# Crystal structure of 8-iodo­quinolinium tetra­chlorido­aurate(III)

**DOI:** 10.1107/S2056989015022574

**Published:** 2015-12-12

**Authors:** Benard O. Onserio, Sem Raj Tamang, James D. Hoefelmeyer

**Affiliations:** a Department of Chemistry, University of South Dakota, 414 E. Clark St., Vermillion, SD 57069, USA

**Keywords:** crystal structure, 8-iodo­quinolinium cation, tetra­chlorido­aurate anion, salt structure

## Abstract

The structure of the title salt, (C_9_H_7_IN)[AuCl_4_], is comprised of planar 8-iodo­quinolinium cations (r.m.s. deviation = 0.05 Å) and square-planar tetra­chlorido­aurate(III) anions. The asymmetric unit contains one 8-iodo­quinolinium cation and two halfs of [AuCl_4_]^−^ anions, in each case with the central Au^III^ atom located on an inversion center. Inter­molecular halogen–halogen contacts were found between centrosymmetric pairs of I [3.6178 (4) Å] and Cl atoms [3.1484 (11), 3.3762 (13), and 3.4935 (12) Å]. Inter­molecular N—H⋯Cl and C—H⋯Cl hydrogen bonding is also found in the structure. These inter­actions lead to the formation of a three-dimensional network. Additionally, there is an intra­molecular N—H⋯I hydrogen bond between the aromatic iminium and iodine. There are no aurophilic inter­actions or short contacts between I and Au atoms, and there are no notable π-stacking inter­actions between the aromatic cations.

## Related literature   

There are only two reported structures containing the 8-iodo­quinolinium cation, *viz.* 8-iodo­quinolinium chloride dihydrate (Son & Hoefelmeyer, 2008*a* ▸) and 8-iodo­quinolinium triiodide tetra­hydro­furan solvate (Son & Hoefelmeyer, 2008*b* ▸). Recently, the zwitterionic 8-iodo­quinoline *N*-oxide was also reported (Hwang *et al.*, 2014 ▸).
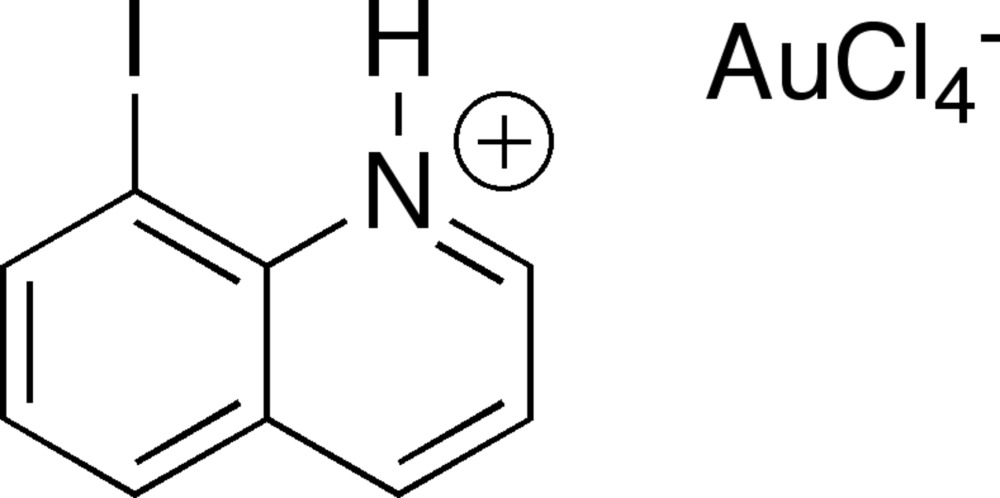


## Experimental   

### Crystal data  

(C_9_H_7_IN)[AuCl_4_]
*M* *_r_* = 594.82
Triclinic, 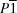

*a* = 7.6299 (5) Å
*b* = 7.8609 (5) Å
*c* = 11.7125 (7) Å
α = 80.160 (1)°
β = 78.143 (1)°
γ = 85.178 (1)°
*V* = 676.52 (7) Å^3^
*Z* = 2
Mo *K*α radiation
μ = 13.92 mm^−1^
*T* = 100 K
0.16 × 0.11 × 0.04 mm

### Data collection  

Bruker APEXII CCD diffractometer
Absorption correction: multi-scan (*SADABS*; Bruker, 2009 ▸) *T*_min_ = 0.174, *T*_max_ = 0.573
6855 measured reflections
2482 independent reflections
2407 reflections with *I* > 2σ(*I*)
*R*_int_ = 0.024

### Refinement  

*R*[*F* ^2^ > 2σ(*F* ^2^)] = 0.016
*wR*(*F* ^2^) = 0.040
*S* = 1.04
2482 reflections
152 parameters
H atoms treated by a mixture of independent and constrained refinement
Δρ_max_ = 1.19 e Å^−3^
Δρ_min_ = −0.94 e Å^−3^

### 

Data collection: *APEX2* (Bruker, 2009 ▸); cell refinement: *SAINT* (Bruker, 2009 ▸); data reduction: *SAINT*; program(s) used to solve structure: *SHELXS97* (Sheldrick, 2008 ▸); program(s) used to refine structure: *SHELXL2014* (Sheldrick, 2015 ▸); molecular graphics: *Mercury* (Macrea *et al.*, 2006 ▸); software used to prepare material for publication: *SHELXTL* (Sheldrick, 2008 ▸).

## Supplementary Material

Crystal structure: contains datablock(s) I, New_Global_Publ_Block. DOI: 10.1107/S2056989015022574/wm5236sup1.cif

Structure factors: contains datablock(s) I. DOI: 10.1107/S2056989015022574/wm5236Isup2.hkl

**Fig. 1.**
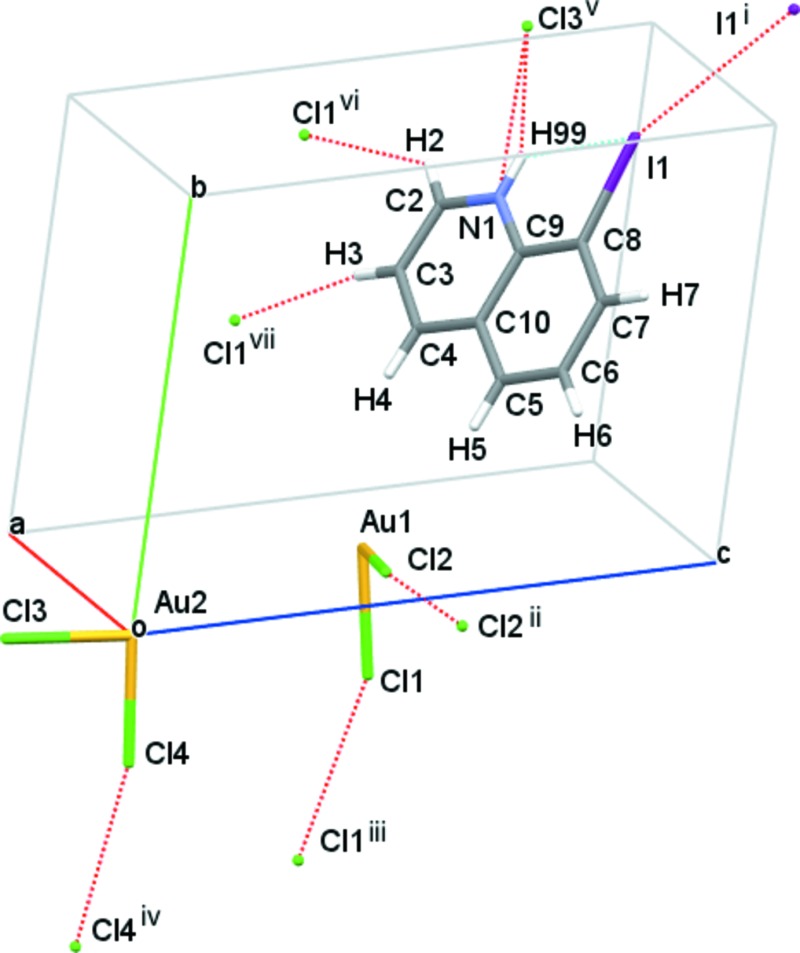
The expanded asymmetric unit of the crystal shown with intermolecular halogen···halogen contacts and hydrogen bonds as dashed lines. [Symmetry codes: (i) 1 − x, 2 − y, 2 − z; (ii) 2 − x, −y, 1 − z; (iii) 1 − x, −1 − y, 1 − z; (iv) −x, −y − 1, −z; (v) x + 1, y + 1, z + 1; (vi) x + 1, y + 1, z; (vii) −x + 2, −y, −z + 1.]

Supporting information file. DOI: 10.1107/S2056989015022574/wm5236Isup3.rtf

x y z x y z x y z x y z x z x y z x y z . DOI: 10.1107/S2056989015022574/wm5236fig1.tif
The expanded asymmetric unit of the crystal shown with inter­molecular halogen⋯halogen contacts and hydrogen bonds as dashed lines. [Symmetry codes: (i) 1 − *x*, 2 − *y*, 2 − *z*; (ii) 2 − *x*, −*y*, 1 − *z*; (iii) 1 − *x*, −1 − *y*, 1 − *z*; (iv) −*x*, −*y* − 1, −*z*; (v) *x* + 1, y + 1, *z* + 1; (vi) *x* + 1, *y* + 1, *z*; (vii) −*x* + 2, −*y*, −*z* + 1.]

**Fig. 2.**
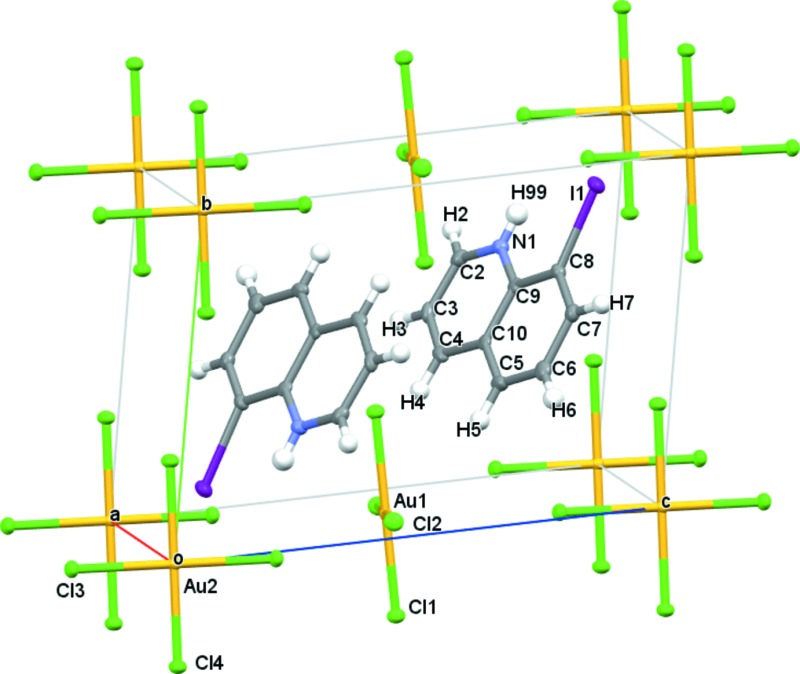
The centrosymmetric unit cell of the title compound. Displacement ellipsoids are drawn at the 50% probability level.

. DOI: 10.1107/S2056989015022574/wm5236fig2.tif
The centrosymmetric unit cell of the title compound. Displacement ellipsoids are drawn at the 50% probability level.

**Fig. 3.**
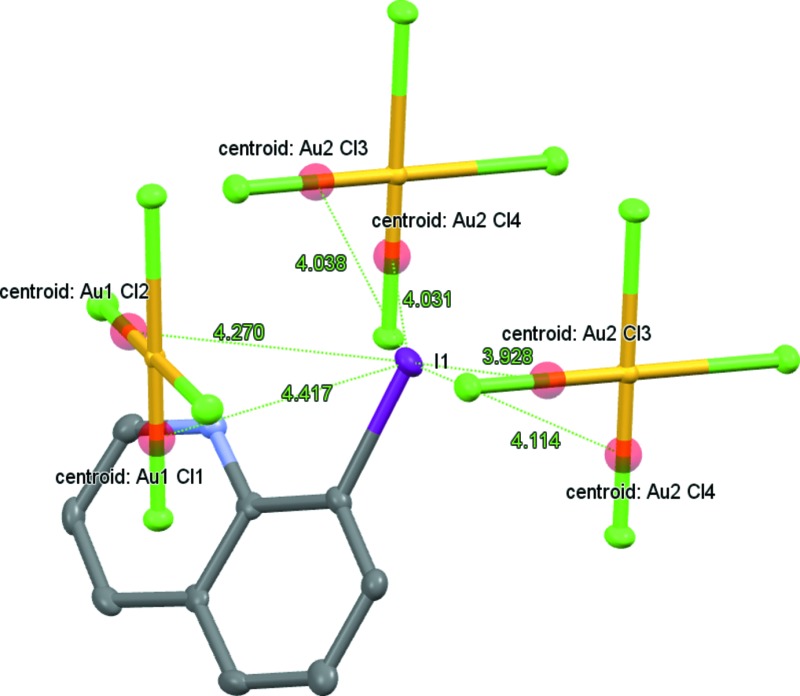
Examination of the nearest distances (Å) between iodine and Au—Cl bond centroids. These distances are beyond the sum of the van der Waals radii of the atoms.

. DOI: 10.1107/S2056989015022574/wm5236fig3.tif
Examination of the nearest distances (Å) between iodine and Au—Cl bond centroids. These distances are beyond the sum of the van der Waals radii of the atoms.

CCDC reference: 1438910

Additional supporting information:  crystallographic information; 3D view; checkCIF report

## Figures and Tables

**Table 1:** Hydrogen-bond geometry (Å, °)

*D*—H⋯*A*	*D*—H	H⋯*A*	*D*⋯*A*	*D*—H⋯*A*
N1—H99⋯Cl3^i^	0.80 (5)	2.62 (5)	3.287 (3)	142 (4)
N1—H99⋯I1	0.80 (5)	2.81 (5)	3.264 (3)	118 (4)
C2—H2⋯Cl1^ii^	0.93	2.79	3.493 (4)	133
C3—H3⋯Cl1^iii^	0.93	2.81	3.722 (4)	168

Symmetry codes: (i) 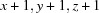; (ii) 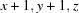; (iii) 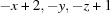.

## supplementary crystallographic information

### S1. Synthesis and crystallization

In a 4 ml vial, HAuCl_4_·3H_2_O (0.12 g, 0.33 mmol), 8-iodo­quinoline (0.10 g, 0.39 mmol) and aceto­nitrile (2 ml) were combined and sonicated for 30 minutes. The 4 ml vial was placed in a 20 ml vial with 5 ml di­ethyl­ether. Diffusion of the ether vapor into the solution within the smaller vial gave yellow-green crystals, mostly with a cuboid-like form.

### S2. Refinement

C-bound H atoms were placed in ideal positions and refined as riding atoms (C—H = 0.93 Å; *U*_iso_(H) = 1.2*U*_eq_(H)). The H atom bound to the N atom was located from a difference map and refined freely. The highest remaining electron density peak was located 0.20 Å from H6. A transmission factor of 0.62 was calculated using the ratio of *T*_min_ (0.4593) to *T*_max_ (0.7452) taken from the absorption correction output file, whereas experimental *T*_min_ (0.174) and *T*_max_ (0.573) give a transmission factor of 0.30.

### Crystal data

**Table:**

(C_9_H_7_IN)[AuCl_4_]	*Z* = 2
*M**_r_* = 594.82	*F*(000) = 536
Triclinic, *P*1	*D*_x_ = 2.920 Mg m^−^^3^
*a* = 7.6299 (5) Å	Mo *K*α radiation, λ = 0.71073 Å
*b* = 7.8609 (5) Å	Cell parameters from 5508 reflections
*c* = 11.7125 (7) Å	θ = 2.6–25.6°
α = 80.160 (1)°	µ = 13.92 mm^−^^1^
β = 78.143 (1)°	*T* = 100 K
γ = 85.178 (1)°	Plate, light green
*V* = 676.52 (7) Å^3^	0.16 × 0.11 × 0.04 mm

### Data collection

**Table:**

Bruker APEXII CCD diffractometer	2407 reflections with *I* > 2σ(*I*)
φ and ω scans	*R*_int_ = 0.024
Absorption correction: multi-scan (*SADABS*; Bruker, 2009)	θ_max_ = 25.4°, θ_min_ = 1.8°
*T*_min_ = 0.174, *T*_max_ = 0.573	*h* = −9→9
6855 measured reflections	*k* = −9→9
2482 independent reflections	*l* = −14→14

### Refinement

**Table:**

Refinement on *F*^2^	0 restraints
Least-squares matrix: full	Hydrogen site location: mixed
*R*[*F*^2^ > 2σ(*F*^2^)] = 0.016	H atoms treated by a mixture of independent and constrained refinement
*wR*(*F*^2^) = 0.040	*w* = 1/[σ^2^(*F*_o_^2^) + (0.019*P*)^2^ + 0.5573*P*] where *P* = (*F*_o_^2^ + 2*F*_c_^2^)/3
*S* = 1.04	(Δ/σ)_max_ = 0.001
2482 reflections	Δρ_max_ = 1.19 e Å^−^^3^
152 parameters	Δρ_min_ = −0.94 e Å^−^^3^

### Special details

**Table:**

Geometry. All e.s.d.'s (except the e.s.d. in the dihedral angle between two l.s. planes) are estimated using the full covariance matrix. The cell e.s.d.'s are taken into account individually in the estimation of e.s.d.'s in distances, angles and torsion angles; correlations between e.s.d.'s in cell parameters are only used when they are defined by crystal symmetry. An approximate (isotropic) treatment of cell e.s.d.'s is used for estimating e.s.d.'s involving l.s. planes.

### Fractional atomic coordinates and isotropic or equivalent isotropic displacement parameters (Å^2^)

**Table:**

	*x*	*y*	*z*	*U*_iso_*/*U*_eq_	
I1	0.54637 (3)	0.86196 (3)	0.88748 (2)	0.01671 (7)	
Au2	0.0000	0.0000	0.0000	0.01009 (6)	
Au1	0.5000	0.0000	0.5000	0.00870 (6)	
Cl3	0.07524 (11)	0.00792 (10)	−0.20000 (7)	0.01714 (17)	
Cl2	0.20893 (10)	0.01686 (10)	0.47699 (7)	0.01590 (17)	
Cl4	0.00376 (12)	−0.29552 (11)	0.02554 (8)	0.01672 (18)	
C8	0.5841 (5)	0.6220 (4)	0.8290 (3)	0.0137 (7)	
C7	0.4426 (5)	0.5185 (5)	0.8453 (3)	0.0167 (8)	
H7	0.3321	0.5507	0.8887	0.020*	
C6	0.4621 (5)	0.3624 (5)	0.7968 (3)	0.0201 (8)	
H6	0.3655	0.2923	0.8092	0.024*	
C5	0.6244 (5)	0.3160 (5)	0.7317 (3)	0.0173 (8)	
H5	0.6359	0.2155	0.6982	0.021*	
C10	0.7737 (5)	0.4170 (4)	0.7144 (3)	0.0136 (7)	
C4	0.9432 (5)	0.3733 (5)	0.6493 (3)	0.0163 (7)	
H4	0.9589	0.2741	0.6142	0.020*	
N1	0.9009 (4)	0.6662 (4)	0.7494 (3)	0.0146 (6)	
C9	0.7541 (5)	0.5716 (5)	0.7656 (3)	0.0134 (7)	
Cl1	0.49806 (11)	−0.29443 (10)	0.53814 (8)	0.01473 (17)	
C3	1.0864 (5)	0.4757 (5)	0.6369 (3)	0.0172 (8)	
H3	1.1983	0.4458	0.5942	0.021*	
C2	1.0612 (5)	0.6228 (5)	0.6887 (3)	0.0166 (8)	
H2	1.1569	0.6925	0.6812	0.020*	
H99	0.890 (6)	0.755 (6)	0.776 (4)	0.026 (12)*	

### Atomic displacement parameters (Å^2^)

**Table:**

	*U*^11^	*U*^22^	*U*^33^	*U*^12^	*U*^13^	*U*^23^
I1	0.01797 (12)	0.01529 (12)	0.01717 (12)	0.00269 (9)	−0.00223 (9)	−0.00651 (9)
Au2	0.01106 (10)	0.00938 (10)	0.01044 (10)	−0.00048 (7)	−0.00355 (7)	−0.00150 (7)
Au1	0.00681 (9)	0.01063 (10)	0.00881 (10)	0.00010 (7)	−0.00208 (7)	−0.00155 (7)
Cl3	0.0245 (4)	0.0160 (4)	0.0109 (4)	−0.0024 (3)	−0.0030 (3)	−0.0017 (3)
Cl2	0.0086 (4)	0.0191 (4)	0.0211 (4)	0.0000 (3)	−0.0055 (3)	−0.0036 (3)
Cl4	0.0233 (4)	0.0104 (4)	0.0166 (4)	−0.0010 (3)	−0.0040 (4)	−0.0020 (3)
C8	0.0163 (17)	0.0134 (17)	0.0117 (17)	0.0026 (14)	−0.0041 (14)	−0.0028 (13)
C7	0.0180 (18)	0.0184 (18)	0.0124 (17)	−0.0002 (14)	−0.0022 (14)	−0.0001 (14)
C6	0.025 (2)	0.0210 (19)	0.0133 (18)	0.0035 (16)	−0.0067 (15)	0.0009 (15)
C5	0.027 (2)	0.0131 (17)	0.0139 (18)	−0.0024 (15)	−0.0092 (15)	−0.0016 (14)
C10	0.0198 (18)	0.0127 (17)	0.0077 (16)	0.0013 (14)	−0.0048 (14)	0.0014 (13)
C4	0.0235 (19)	0.0133 (17)	0.0130 (17)	0.0058 (14)	−0.0080 (15)	−0.0021 (14)
N1	0.0164 (15)	0.0125 (15)	0.0153 (15)	0.0006 (12)	−0.0041 (12)	−0.0024 (12)
C9	0.0177 (17)	0.0125 (17)	0.0098 (16)	0.0008 (13)	−0.0067 (14)	0.0027 (13)
Cl1	0.0150 (4)	0.0115 (4)	0.0176 (4)	−0.0005 (3)	−0.0037 (3)	−0.0014 (3)
C3	0.0138 (17)	0.0234 (19)	0.0117 (17)	0.0055 (15)	−0.0005 (14)	−0.0007 (14)
C2	0.0159 (18)	0.0173 (18)	0.0159 (18)	−0.0029 (14)	−0.0053 (14)	0.0026 (14)

### Geometric parameters (Å, º)

**Table:**

I1—C8	2.093 (3)	C6—H6	0.9300
Au2—Cl3	2.2857 (8)	C5—C10	1.404 (5)
Au2—Cl3^i^	2.2857 (8)	C5—H5	0.9300
Au2—Cl4^i^	2.2894 (8)	C10—C4	1.407 (5)
Au2—Cl4	2.2895 (8)	C10—C9	1.429 (5)
Au1—Cl1^ii^	2.2817 (8)	C4—C3	1.381 (5)
Au1—Cl1	2.2817 (8)	C4—H4	0.9300
Au1—Cl2^ii^	2.2818 (8)	N1—C2	1.331 (5)
Au1—Cl2	2.2818 (8)	N1—C9	1.360 (5)
C8—C7	1.369 (5)	N1—H99	0.80 (4)
C8—C9	1.418 (5)	C3—C2	1.377 (5)
C7—C6	1.422 (5)	C3—H3	0.9300
C7—H7	0.9300	C2—H2	0.9300
C6—C5	1.371 (5)		
			
Cl3—Au2—Cl3^i^	180.0	C6—C5—C10	121.4 (3)
Cl3—Au2—Cl4^i^	90.15 (3)	C6—C5—H5	119.3
Cl3^i^—Au2—Cl4^i^	89.85 (3)	C10—C5—H5	119.3
Cl3—Au2—Cl4	89.85 (3)	C5—C10—C4	123.3 (3)
Cl3^i^—Au2—Cl4	90.15 (3)	C5—C10—C9	118.8 (3)
Cl4^i^—Au2—Cl4	180.0	C4—C10—C9	117.9 (4)
Cl1^ii^—Au1—Cl1	180.0	C3—C4—C10	120.9 (3)
Cl1^ii^—Au1—Cl2^ii^	90.54 (3)	C3—C4—H4	119.6
Cl1—Au1—Cl2^ii^	89.46 (3)	C10—C4—H4	119.6
Cl1^ii^—Au1—Cl2	89.46 (3)	C2—N1—C9	123.7 (3)
Cl1—Au1—Cl2	90.54 (3)	C2—N1—H99	118 (3)
Cl2^ii^—Au1—Cl2	180.0	C9—N1—H99	118 (3)
C7—C8—C9	119.9 (3)	N1—C9—C8	122.7 (3)
C7—C8—I1	120.2 (3)	N1—C9—C10	117.9 (3)
C9—C8—I1	119.8 (3)	C8—C9—C10	119.4 (3)
C8—C7—C6	121.0 (3)	C2—C3—C4	119.0 (3)
C8—C7—H7	119.5	C2—C3—H3	120.5
C6—C7—H7	119.5	C4—C3—H3	120.5
C5—C6—C7	119.5 (4)	N1—C2—C3	120.6 (3)
C5—C6—H6	120.3	N1—C2—H2	119.7
C7—C6—H6	120.3	C3—C2—H2	119.7

Symmetry codes: (i) −*x*, −*y*, −*z*; (ii) −*x*+1, −*y*, −*z*+1.

### Hydrogen-bond geometry (Å, º)

**Table:**

*D*—H···*A*	*D*—H	H···*A*	*D*···*A*	*D*—H···*A*
N1—H99···Cl3^iii^	0.80 (5)	2.62 (5)	3.287 (3)	142 (4)
N1—H99···I1	0.80 (5)	2.81 (5)	3.264 (3)	118 (4)
C2—H2···Cl1^iv^	0.93	2.79	3.493 (4)	133
C3—H3···Cl1^v^	0.93	2.81	3.722 (4)	168

Symmetry codes: (iii) *x*+1, *y*+1, *z*+1; (iv) *x*+1, *y*+1, *z*; (v) −*x*+2, −*y*, −*z*+1.
