# Supplementary material for: Crystal structure of 8-iodo­quinolinium tetra­chlorido­aurate(III)
Source: Acta Crystallogr E Crystallogr Commun. 2015 Dec 12;71(Pt 12):m261–2. doi: 10.1107/S2056989015022574 (PMC4719864; doi:10.1107/S2056989015022574)
Supplement: Supplementary file 3 [file e-71-0m261-Isup3.rtf]

Structural commentary The centrosymmetric unit cell (figure 1) contains two 8-iodoquinolinium cations.  The corner positions and two face-center positions of the unit cell are occupied by tetrachloroaurate(III) anions.  The tetrachloroaurate(III) anions do not associate via aurophilic interaction.  The cations do not exhibit pi-stacking interactions.  The 8-iodoquinolinium cation has six nearest neighbor tetrachloroaurate anions.  The closest contacts between the cation and anion are via N-H...Cl (2.618 \%A) and C-H...Cl (ca 2.97 \%A) (figure 2) that fall near the sums of the Van der Waals radii. In figure 3, nearest iodine to Au-Cl bond centroids are shown.  These distances are beyond the sum of the Van der Waals radii of the atoms. ynthesis and crystallizationIn a 4 mL vial, HAuCl~4~.3H~2~O (0.12 g, 0.33 mmol), 8-iodoquinoline (0.10 g, 0.39 mmol) and acetonitrile (2 mL) were combined and sonicated for 30 minutes.  The 4 mL vial was placed in a 20 mL vial with 5 mL diethylether. Diffusion of the ether vapor into the solution within the smaller vial gave yellow-green cubic crystals. 
