# Supplementary figures and images for: Crystal structure of 8-iodo­quinolinium tetra­chlorido­aurate(III)
Source: Acta Crystallogr E Crystallogr Commun. 2015 Dec 12;71(Pt 12):m261–2. doi: 10.1107/S2056989015022574 (PMC4719864; doi:10.1107/S2056989015022574)

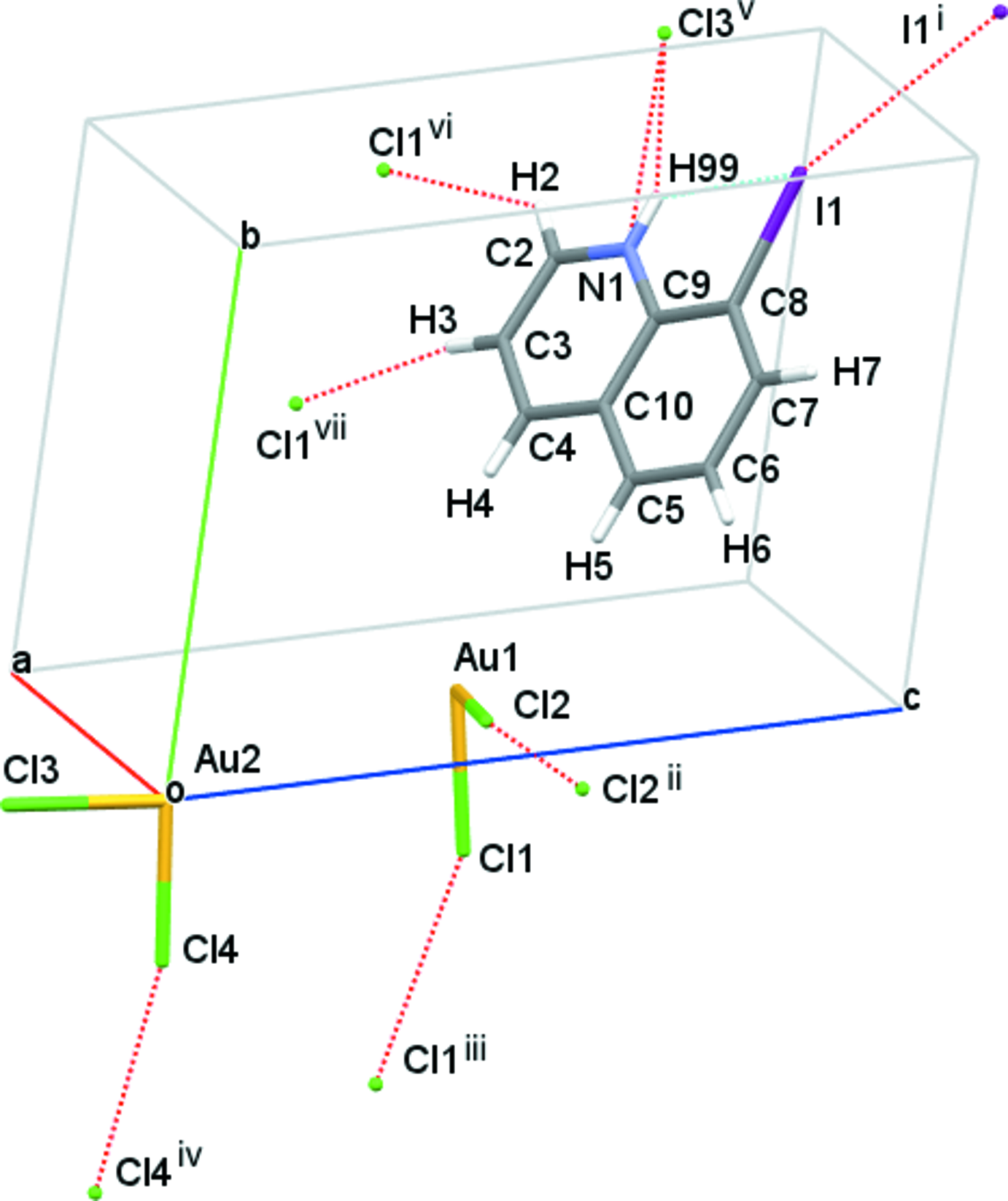

Supplement: Supplementary file 4 [file e-71-0m261-fig1.tif]

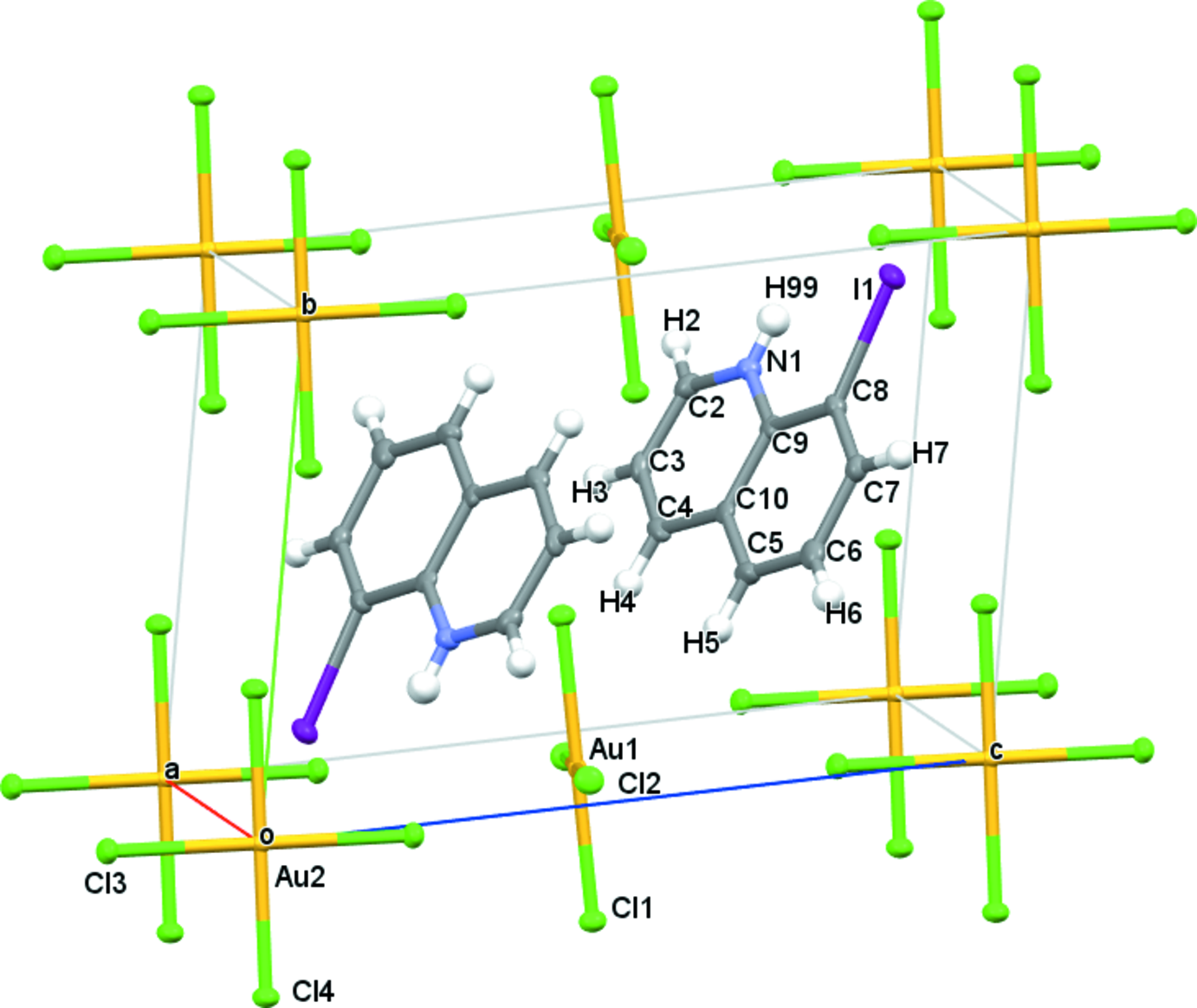

Supplement: Supplementary file 5 [file e-71-0m261-fig2.tif]

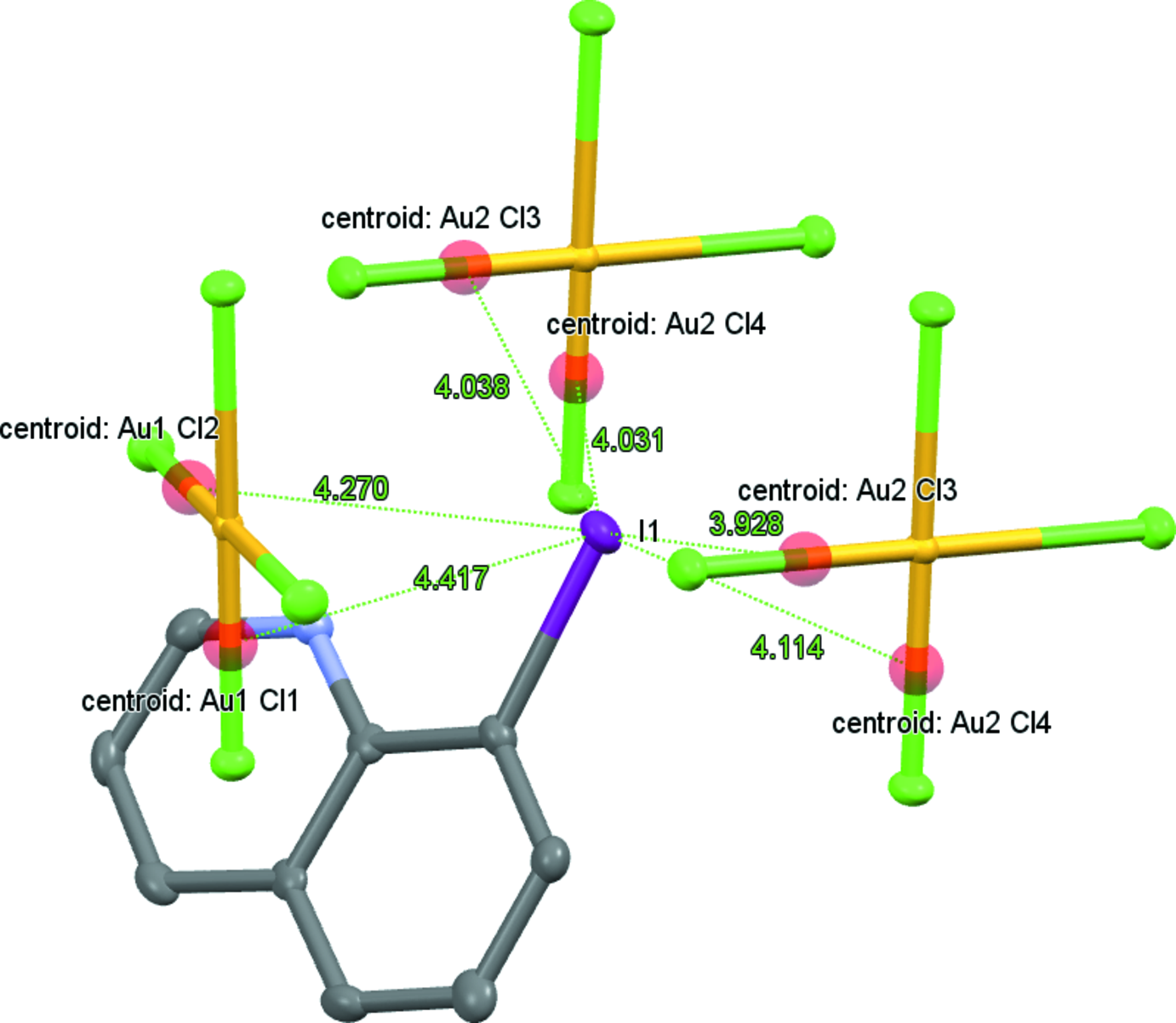

Supplement: Supplementary file 6 [file e-71-0m261-fig3.tif]
